# Building bridges between public health and computer science departments in academia to foster artificial intelligence knowledge and skills for the digital age

**DOI:** 10.3389/fpubh.2026.1871844

**Published:** 2026-07-27

**Authors:** Seble Frehywot, Michael Cowling, Evangelos Pappas, Yianna Vovides

**Affiliations:** 1 Department of Global Health, George Washington University, Washington, DC, United States; 2 Co-Founder IT for Health and Education Systems Equity (ITfHESE), Washington, DC, United States; 3 Hub for Apple Platform Innovation (HAPI), School of Computing Technologies, STEM College, Royal Melbourne Institute of Technology (RMIT) University, Melbourne, VIC, Australia; 4 Australasian Society for Computers in Learning in Tertiary Education, Tugun, QLD, Australia; 5 Education/Information and Communication Technology, Central Queensland University, Norman Gardens, QLD, Australia; 6 School of Health and Biomedical Sciences, MedTech Research and Innovation Hub, Royal Melbourne Institute of Technology (RMIT) University, Melbourne, VIC, Australia; 7 Shanghai University of Medicine and Health Sciences, Pudong, Shangai, China; 8 Centers in New Design in Learning and Scholarship, Georgetown University, Washington, DC, United States

**Keywords:** academic bridging, artificial intelligence, digital public health, interdisciplinary collaboration, public health and computer science

## Abstract

The current communication gap between Public Health (PH) and Computer Science (CS) academics is striking: public health professionals speak in terms of incidence rates, risk factors, interventions, health systems, health policy, and equity, while computer scientists converse in neural networks, optimization functions, scalability, and computational complexity. This linguistic and cultural divide often prevents natural collaboration, leaving valuable opportunities unexplored. In this Artificial Intelligence (AI) era, regardless of whether they are in high-, middle-, or low-income countries, these two departments stand to gain immensely from forging a simple yet powerful bridge between themselves. Connecting through joint seminars, cross-listed courses, shared research projects, co-mentorship of students, and informal faculty and staff exchanges offers clear, immediate advantages: accelerated innovation in AI applications for public health challenges, enhanced employability for graduates entering a job market hungry for "AI-savvy" health professionals, and the cultivation of ethical, context-aware AI solutions that truly serve populations rather than merely advancing technology. Yet overcoming institutional barriers such as siloed budgets, geographical barriers (different buildings, districts, or campuses), differing promotion criteria, or scheduling conflicts and cultural ones, such as mutual unfamiliarity with each other's priorities, demands deliberate but feasible strategic planning. This perspective article articulates these benefits and challenges and then concludes with a set of strategies, proposed by a group of public health, health, and computer science academics, to break this divide and build a bridge between PH and CS.

## 1 Introduction

**Figure 1:**
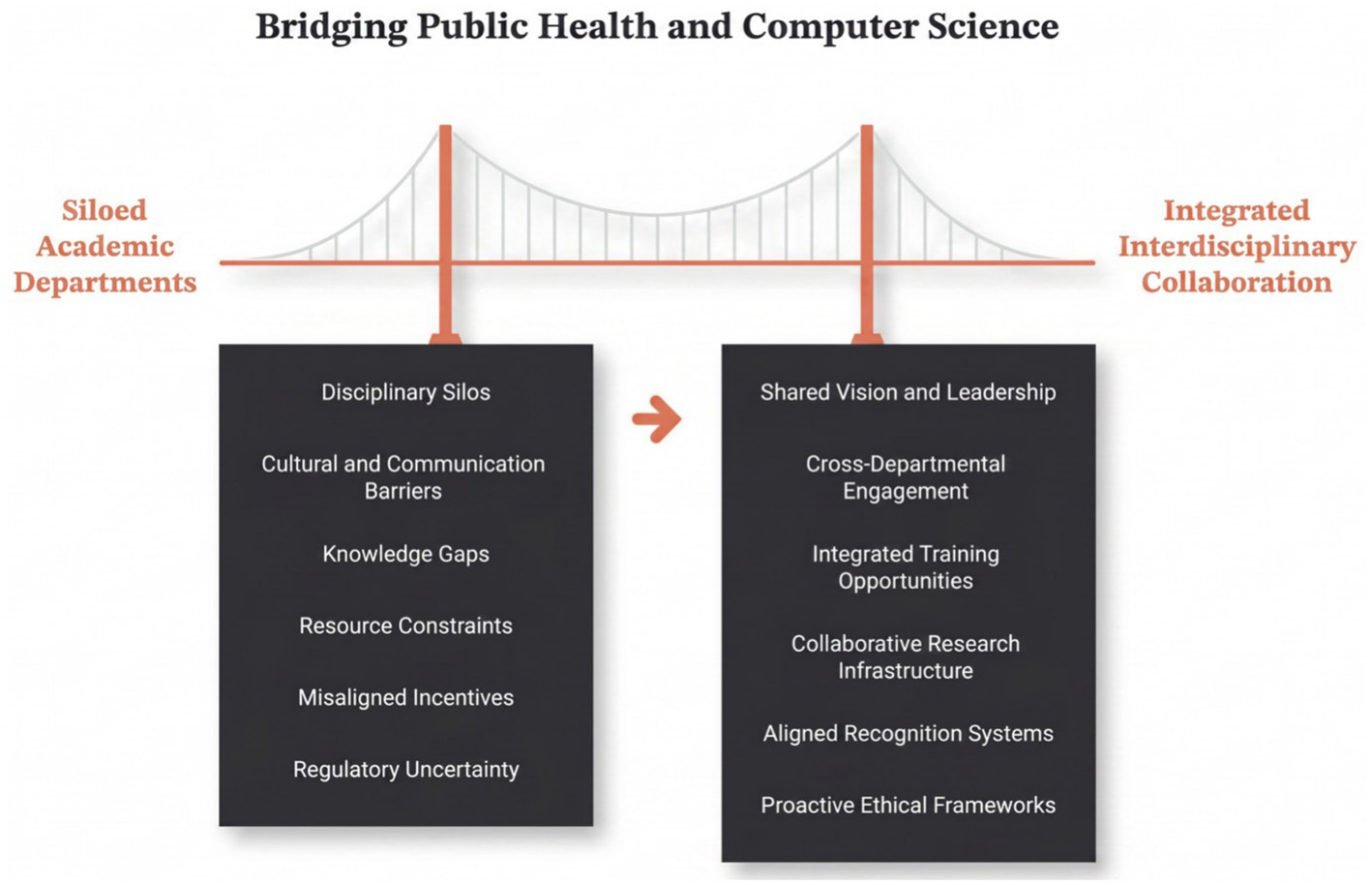
Strategies for building bridges between public health and computer science departments in academia to foster ai knowledge & skills for the digital age.

In the rapidly evolving digital age, where Artificial Intelligence (AI) is swiftly transforming every sector of society, academic institutions worldwide, regardless of whether they are in high-, middle-, or low-income countries, stand to gain immensely from forging a simple yet *powerful bridge* between their Public Health and Computer Science departments (Figure 1). Public Health schools train the next generation of professionals to tackle epidemics, health inequities, chronic diseases, and health system challenges, and are increasingly confronted with vast amounts of complex data that demand sophisticated analytical tools (1).

At the same time, Computer Science departments, rich in expertise in algorithms, machine learning, data structures, and AI model development, often lack a meaningful mechanism by which to collaborate within real-world, high-impact application domains where their innovations can directly improve human wellbeing. (2) Creating interdisciplinary connections between these two fields represents one *of the lowest-hanging fruits not only in academia but also in health systems*: it requires no massive new infrastructure or competitive external grants, and no radical restructuring. Instead, it leverages existing faculty, students, curricula, and geographical proximity to spark mutual learning (3). Public Health students and faculty can quickly gain practical AI skills—such as predictive modeling for disease outbreaks, natural language processing for analyzing health records, or computer vision for diagnostic support, while Computer Science counterparts acquire domain knowledge in epidemiology, health policy, global health, health systems, ethics of data use, and the social determinants of health. This exchange enriches both sides, turning abstract technical prowess into a tangible public good and grounding public health practice in cutting-edge computational capabilities.

Since 2020, our global research initiative has focused on building bridges between global public health, health, and AI communities and establishing an international network of Communities of Practice (4). Hundreds of research universities worldwide maintain both public health and computer science departments. Public Health departments may function as standalone departments, as independent colleges within the university, or as units housed within a school of medicine or health sciences. Similarly, Computer Science departments may operate as standalone departments or as components of a school of engineering. Many public health programs lack dedicated sections in AI informatics, data science, or biostatistics, and they frequently encounter challenges in initiating AI education and training for students and faculty. This effort highlights a substantial but underutilized opportunity for cross-disciplinary engagement. In such contexts, leveraging proximate computer science departments represents a pragmatic and accessible pathway to address these needs, while enabling computer science programs to expand their applied research and educational outputs in public health domains through ethically grounded, mutually beneficial partnerships.

In simple terms, we propose better connections and collaborations between the computer scientists who have technical solutions and the public health professionals who are intimately familiar with the most urgent priorities in public health and healthcare.

This perspective paper outlines the substantial benefits of such bridges, the common challenges encountered across diverse national contexts, and a set of practical, actionable steps that universities can adopt to initiate and sustain meaningful interdisciplinary collaboration in AI for public health. By taking this low-effort, high-reward path, academic institutions can position themselves at the forefront of preparing professionals equipped to harness AI responsibly for healthier societies worldwide in this AI era.

## 2 Integration with global evidence and policy frameworks

The manuscript incorporates a broader evidence base to situate the proposed framework within current global knowledge. The WHO Global Strategy on Digital Health (5) underscores the need for capacity building and people-centered digital systems. This strategy emphasizes strengthening health systems through accessible, scalable, and sustainable digital health solutions while promoting global collaboration, national strategy development, robust governance, and workforce competencies in digital health. It explicitly calls for integrating digital health literacy and skills into education and training curricula for health professionals at all levels, aligning closely with the framework's focus on bidirectional Public Health and Computer Science departments' capacity building.

In addition, the WHO guidance on Ethics and Governance of Artificial Intelligence for Health (6), along with its 2025 extension on large multi-modal models (LMMs) (7), and the WHO Global strategy on digital health (LMM) (8), outline six core principles — autonomy, wellbeing, justice, equity, non-maleficence, and transparency — and provide actionable recommendations for addressing bias, fairness, privacy, and accountability in AI deployment for health. The 2025 LMM guidance specifically examines the benefits and risks of generative AI in health applications, reinforcing the manuscript's emphasis on embedding ethical governance from the outset of Public Health and Computer Science departments collaborations.

Further support is drawn from OECD reports on Scaling AI in Health, AI maturity across countries, and workforce transformation (9), as well as on AI literacy, digital health education frameworks, interdisciplinary collaboration models, and responsible AI implementation (29) These reports highlight significant gaps in AI skills within the health workforce and stress the importance of responsible scaling, data governance, and interdisciplinary approaches to realize AI's potential while mitigating risks. They document varying levels of AI maturity among OECD countries — with most at an emerging stage — and underscore the need for targeted training to build digital and AI competencies, directly supporting the framework's low-barrier, education-focused bridge-building steps.

These integrations strengthen the manuscript's alignment with international standards and enhance its relevance for implementation across diverse resource settings.

## 3 Benefits of interdisciplinary public health & computer science departments' collaboration

Interdisciplinary approaches have been shown to accelerate innovation, as demonstrated by successful AI applications during the COVID-19 pandemic and in ongoing disease surveillance systems. Case studies of interdisciplinary teams consistently yield higher-impact outputs, particularly in the development and deployment of AI tools for epidemic response (10, 11). Such collaborations also enhance graduate employability by equipping students with combined technical and domain-specific expertise. Employers in health data science increasingly seek professionals who possess both advanced computational skills and a deep understanding of public health contexts, and interdisciplinary AI training directly strengthens skills diversity and workforce readiness (12).

Furthermore, targeted training programs within these partnerships increase AI readiness across the public health workforce. They directly address documented skill gaps identified in OECD analyses, thereby closing critical deficiencies in health-sector AI competencies (13). Finally, interdisciplinary teams have been linked to superior patient and population-level results across multiple studies, including enhanced epidemic surveillance, reduced health disparities, and optimized public health responses (14–16).

## 4 Challenges: for planning the bridge

While building bridges between Public Health & Computer Science in academia to foster AI knowledge & skills in the digital Age is essential, it needs to be approached with awareness of the several challenges.

### 4.1 Disciplinary silos

Public Health and Computer Science departments often operate in isolation with distinct research priorities, methodologies, and terminologies, leading to limited understanding of each other's domains (17). Faculty may lack familiarity with the potential of AI in public health or the public health problems that Computer Science can address (18). Some clinicians may even be cautious, negatively predisposed, or even scared and threatened by the rapid adoption of AI in academia and healthcare, and remain on the sidelines of the current revolution.

### 4.2 Cultural and communication barriers

Differences in academic cultures (e.g., applied vs. theoretical focus) and jargon can hinder effective collaboration. Public Health faculty may view AI as a “black box,” while Computer Science faculty may not fully grasp the contextual nuances of public health data and problems (19) or know how to identify public health areas that are conducive to disruption by computer technology advances.

### 4.3 Resource constraints

There is limited funding, time, or institutional support for interdisciplinary initiatives, with the academy often represented as being made up of separate silos that each conduct their own work (20). Lack of infrastructure (e.g., shared computational resources, data platforms) to support collaborative AI projects adds to the challenge.

### 4.4 Misaligned incentives

Faculty are often evaluated based on publications and grants within their own fields, discouraging interdisciplinary work. Tenure and promotion criteria may not reward collaborative efforts (21). Even though interdisciplinary work has become more popular and, in many universities, strongly encouraged and incentivized, career progression criteria frequently lag behind.

### 4.5 Knowledge gaps

Public Health staff may lack technical expertise in AI, while Computer Science faculty may not understand public health challenges (e.g., epidemiology, health equity, health policy, health systems). Both disciplines suffer from a high level of technical language that can be difficult to translate across the discipline divide. This may also be compounded by the limited awareness of ethical considerations in AI for public health (e.g., bias, privacy).

### 4.6 Data access and integration

Public health datasets are often sensitive, fragmented, or governed by strict regulations, complicating their use in AI research. Lack of standardized data formats or interoperable systems between departments also raises issues.

### 4.7 Sustainability

Initial collaborations may fizzle out without long-term institutional commitment or funding, which presents difficulty in maintaining momentum for interdisciplinary programs.

## 5 Strategies: for building the bridge

For building a bridge between the Public Health and Computer Science Departments, *a bridge has to be built with the intention* of addressing the above challenges, moving from siloed academic departments to an integrated interdisciplinary collaboration. This requires definitive strategies (Figure 1) that form a sequence of planks being built out from both sides of our bridge, such that they will meet in the middle.

### 5.1 Central contribution of the manuscript: bridge building framework

**Figure 2:**
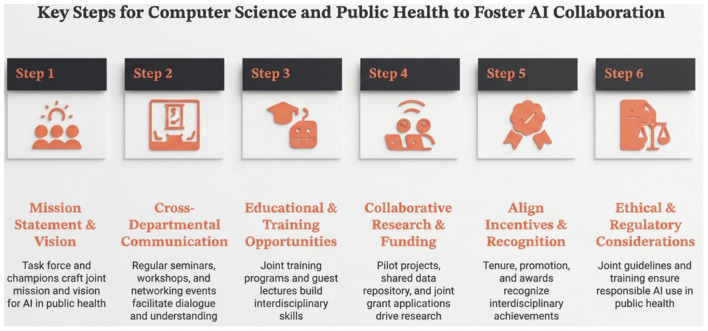
“Bridge-building” framework specifically designed to foster artificial intelligence collaboration between public health and computer science departments within academic institutions.

The central contribution of this manuscript is a pragmatic, six-step “bridge-building” framework specifically designed to foster artificial intelligence (AI) collaboration between Public Health (PH) and Computer Science (CS) departments within academic institutions (Figure 2). The *novelty* of this framework lies in its pragmatic, low-barrier “*bridge-building”* approach. It employs a reciprocal “*planks from both sides”* metaphor that emphasizes mutual, bidirectional contributions—each discipline builds its own planks—while relying exclusively on existing departmental resources without necessitating major new infrastructure or competitive external grants (5, 30).

The framework operationalizes a clear, sequential yet reciprocal process—shared vision, communication, education, research, incentives, ethics, and sustainability—tailored explicitly to Public Health and Computer Science departments' AI collaboration. This structure directly addresses the unique demands of the AI era: Public Health requires rapid technical upskilling, while Computer Science benefits from domain grounding in epidemiology, ethics, social determinants of health, and real-world data applications.

This framework distinguishes itself from broader interdisciplinary models by foregrounding mutual, bidirectional capacity-building in the specific context of the AI era. Unlike more general health informatics models, the framework prioritizes “*opportunistic impact”* through faculty champions, integrates ethical governance from the outset, and offers low-effort entry points, such as shared glossaries and pilot projects focused on outbreaks or health equity (31). It emphasizes opportunistic impact via champions and early, low-effort activities, and integrates ethical governance from the outset. In doing so, it aligns closely with—but operationalizes for academic departments—the World Health Organization's guidance for resource-variable settings spanning high-, middle-, and low-income countries (6, 7).

In general, interdisciplinary approaches in translational research often presuppose top-down funding mechanisms or dedicated centers. In contrast, this framework adopts a bottom-up strategy that leverages existing resources and explicitly mitigates AI-specific barriers, including “black box” perceptions and concerns around data sensitivity (6). Its unique contribution to Digital Public Health scholarship is a concrete, actionable roadmap that positions academic departmental bridges as the foundational “*lowest-hanging fruit”* for responsible AI deployment. It offers a practical pathway for building AI literacy and enabling responsible deployment in resource-variable settings, thereby aligning with and extending WHO strategies through a focused emphasis on departmental collaboration (8). Suitability for the AI era stems from the fact that many earlier approaches predate the widespread adoption of generative AI and large multimodal models. This framework integrates ethics and governance early (consistent with WHO 2021/2025 guidance) and stresses long-term sustainability within a rapidly evolving technological landscape (31).

### 5.2 Components of the bridge

#### 5.2.1 Establish a shared vision and leadership

To build a strong bridge between the Public Health and Computer Science departments, the first essential step is to establish a shared vision and committed leadership. This begins *by tackling the common barriers of disciplinary silos and misaligned incentives head-on* through forming an interdisciplinary task force composed of academic members from both departments, tasked with collaboratively defining clear, mutually beneficial goals, such as advancing AI-driven public health solutions in priority areas, such as real-time disease surveillance, predictive modeling for outbreaks, or health equity analysis. The priority areas will need to be defined based on the public health priorities and the areas of expertise of departments where there is a critical mass for fruitful collaborations.

To drive momentum, dedicated champions should be appointed, ideally faculty who already possess cross-disciplinary experience, to actively advocate for the collaboration, help align departmental priorities, and navigate potential resistance. It's in building these first planks onto the bridge on both sides, facilitated by passionate faculty members who are keen to collaborate, that we establish a foundation for future steps, referred to as *opportunistic impact* (22). Identifying or hiring a champion with (preferably) dual expertise, e.g., a computer scientist with a Master's in Public Health, would catalyze this initiative.

Together, the task force and champions should craft a compelling joint mission statement that highlights the profound societal impact of integrating AI with public health, such as improving overall health outcomes, reducing disparities, and addressing pressing global challenges. This foundational step can culminate in a unifying vision, for instance, the creation of a joint “AI for Public Health” research center that provides a clear road map and inspires sustained commitment from both sides.

#### 5.2.2 Facilitate cross-departmental communication

The second key step in bridging Public Health and Computer Science departments is to facilitate robust cross-departmental communication. This can be achieved by hosting regular seminars, workshops, and journal clubs where faculty from both sides present their ongoing work and collaboratively explore promising AI applications in public health, such as predictive modeling for pandemics or outbreak forecasting. Additionally, organizing informal “speed networking” events can help build personal relationships and quickly identify overlapping research interests between the two communities. Starting with presentations that demonstrate the success of combined computer science and public health approaches in solving major health problems, resulting in high-profile publications and/or grant funding, will create enthusiasm and momentum.

To overcome technical language barriers, the groups should jointly develop a shared glossary of key terms—translating concepts like “machine learning” and “deep neural networks” from Computer Science into accessible language while introducing Public Health terms such as “social determinants of health” and “health equity metrics” to their computer science colleagues. *By directly addressing cultural and communication barriers through these activities*, for example, having Computer Science faculty explain AI techniques and their applications in plain terms while Public Health experts present pressing real-world health challenges, this step creates an environment of mutual understanding and lays the groundwork for meaningful, ongoing collaboration.

#### 5.2.3 Build educational and training opportunities

The third critical step is to build educational and training opportunities *by directly addressing knowledge gaps* and cultivating the next generation of interdisciplinary researchers. This involves creating joint training programs, such as short courses or certificate programs focused on AI for public health, covering topics like machine learning applications in epidemiological modeling, responsible data use, and ethical considerations in health AI. To foster mutual understanding, Computer Science faculty should be encouraged to give guest lectures in Public Health courses to explain technical AI concepts in accessible ways, while Public Health faculty reciprocate by introducing real-world health challenges, data contexts, and ethical frameworks in Computer Science classes.

Over time, departments can develop more ambitious interdisciplinary graduate programs or specialized tracks, such as an MS or PhD in AI and Public Health, designed to train students who are equally fluent in both domains. Appointing conjoint academics with dual appointments in Public Health and Computer Science will accelerate these collaborations. These efforts can be exemplified by co-taught workshops where Computer Science and Public Health faculty jointly guide participants through hands-on projects, such as using AI to analyze complex public health datasets, thereby equipping students and academics alike with the integrated skills needed for impactful collaborations.

#### 5.2.4 Foster collaborative research projects and secure funding and resources

The fourth key step focuses on fostering collaborative research projects that translate shared vision into concrete action and securing possible funding and resources. This can begin with initiating small-scale pilot projects that deliberately require expertise from both sides, such as developing AI models to predict disease outbreaks, identify emerging health disparities, or analyze the complex interplay of social determinants of health. To enable these efforts, the departments should collaboratively create a secure, shared data repository containing de-identified public health datasets, carefully designed to comply with regulations like HIPAA while giving computer science researchers accessible, high-quality data for training and testing AI algorithms. Building on this foundation, interdisciplinary research groups or working teams can be established to focus on targeted challenges, for example, optimizing AI-driven vaccine distribution strategies or modeling the impact of public health interventions. *By directly addressing barriers related to data access, integration*, and lingering disciplinary silos, this step generates early successes, builds trust, and creates momentum for larger, more ambitious joint research endeavors. This can then segway for Public Health and Computer Science departments to secure funding and resources that can sustain meaningful collaboration with actively pursuing joint grant applications targeting agencies and foundations that prioritize interdisciplinary work at the intersection of AI and public health, such as those supporting research in disease modeling, health equity, or pandemic preparedness. At the same time, departments should advocate internally for institutional support to create shared infrastructure, including a dedicated data science lab, high-performance computing resources, or secure cloud environments tailored for collaborative projects involving sensitive health data.

To complement these efforts, forging partnerships with industry partners and public health organizations can provide additional funding for pilot projects—such as developing AI tools for contact tracing, health policy simulation, or equitable resource allocation—demonstrating tangible value and attracting further investment. *By directly addressing resource constraints* through these coordinated strategies, the two departments can build the financial and technical foundation necessary to turn shared vision and ideas into sustained, high-impact research and innovation.

#### 5.2.5 Align incentives and recognition

The fifth vital step is to align incentives and recognition systems so that interdisciplinary collaboration is genuinely rewarded rather than penalized. This requires working closely with university leadership to revise tenure and promotion criteria, ensuring that achievements such as co-authored publications in high-impact journals, successful joint grant proposals, and cross-departmental mentoring are explicitly valued and counted toward career advancement in both fields. At the same time, successful collaborations should be actively highlighted through university-wide channels, such as awards, featured stories in newsletters, and public events, to visibly celebrate progress and motivate broader participation. Departments can further strengthen ties by creating joint appointments or affiliate faculty positions that formally recognize and institutionalize collaboration between the two units. *By addressing the persistent challenge of misaligned incentives in this way*, for instance, treating a groundbreaking joint AI-public health publication as a significant achievement for both departments, this step helps shift the academic culture toward one that actively supports and sustains long-term interdisciplinary engagement.

#### 5.2.6 Address ethical and regulatory considerations

The sixth essential step is to proactively address ethical and regulatory considerations that underpin responsible interdisciplinary work. This involves jointly developing clear guidelines for the ethical use of AI in public health, with input from both departments, to tackle critical issues such as algorithmic bias, model transparency, fairness in health equity applications, and robust data privacy protections. Faculty and researchers from both sides should receive targeted training on key regulatory requirements, including Institutional Review Board (IRB)/Ethics processes, data-sharing agreements, and compliance with health privacy standards, ensuring that collaborative projects can move forward smoothly and ethically. *By directly confronting governance and privacy issues around ethics and data access*, this step builds confidence and reduces potential roadblocks. A practical example is the creation of a shared checklist or framework for AI-public health projects that helps teams systematically evaluate and uphold ethical and legal standards from the outset, fostering trust and enabling high-quality, responsible research that truly serves society.

## 6 Implementation

To translate the identified challenges and corresponding strategies into actionable progress, a phased implementation approach is recommended.

### 6.1 Possible implementation timelines

This structured timeline leverages incremental milestones to foster sustainable collaboration between Public Health and Computer Science departments, ultimately advancing AI knowledge, skills, and impactful research in the digital age.

In the initial 0–6 months, institutions should prioritize foundational activities: forming an interdisciplinary task force comprising faculty and administrators from both departments, hosting initial seminars and workshops to build awareness and dialogue, identifying potential pilot projects that align with shared research interests, and submitting applications for seed funding to support early initiatives.

During the 6–12 months phase, efforts shift toward operationalizing collaboration. This includes launching joint training programs for faculty and students, initiating selected pilot projects, and establishing formal data-sharing protocols that address privacy, security, and ethical considerations inherent to public health applications of AI.

From 1–2 years, the focus moves to scaling and institutionalization. Key activities encompass securing major external grants, formalizing interdisciplinary academic programs or certificate tracks, and advocating for broader institutional policy changes—such as revised promotion and tenure criteria that recognize cross-disciplinary contributions.

Beyond 2 years, the initiative matures into long-term structural change. This stage involves establishing a dedicated center or institute for AI in Public Health, conducting comprehensive outcome evaluations, and expanding collaborations to include external partners such as industry, government agencies, and other academic institutions.

### 6.2 Embedded evaluation framework and metrics for success

An embedded evaluation framework, drawing from established models such as Kirkpatrick's four levels of training evaluation (reaction, learning, behavior, and results) and translational AI evaluation approaches like the Translational Evaluation of Healthcare AI (TEHAI), provides a robust mechanism for assessing progress. This framework emphasizes both process and outcome indicators across short-, medium-, and long-term horizons.

Success can be measured through the following key indicators:

• *Number of joint publications, grants, and collaborative projects* initiated between the two departments.

• *Faculty participation rates* in interdisciplinary events, training sessions, and co-teaching opportunities.

• *Student enrollment and completion rates* in newly developed interdisciplinary tracks or courses.

• *Development and utilization of shared resources*, including integrated data platforms, computing laboratories, and methodological toolkits.

• *Ethical compliance rates*, such as adherence to established AI ethics guidelines and successful institutional review board approvals for joint projects.

• *Measurable improvements in public health outcomes*, including enhancements in health equity metrics, disease surveillance capabilities, or intervention effectiveness attributable to AI applications developed through the collaboration.

### 6.3 Causal pathways and outcome horizons

The pathway from challenge to impact follows a logical causal sequence: *targeted strategies* (e.g., shared training, resource pooling, and policy advocacy) address *specific challenges* (such as cultural differences, resource constraints, and siloed expertise), leading to *intermediate outcomes* like increased trust, mutual understanding, and technical interoperability. These, in turn, drive *long-term impacts*, including sustained interdisciplinary capacity and tangible advancements in AI-driven public health solutions.

Anticipated outcomes are tiered as follows:

• **Short-term**: Successful pilot projects, development of a shared glossary of terms, and initial cross-departmental networks.

• **Medium-term**: Joint grants and publications, formalized student training tracks, and operational shared infrastructure.

• **Long-term**: Establishment of a dedicated center, scalable models for other institutions, and demonstrable contributions to health equity and population health improvement.

By systematically addressing structural, cultural, and resource-related challenges through strategic collaboration, shared infrastructure, and sustained institutional support, Public Health and Computer Science departments can effectively bridge disciplinary divides. This integration will not only advance AI knowledge and skills within academia but also position institutions to generate transformative research that addresses pressing public health needs in the AI era.

### 6.4 Critical implementation considerations

In addition to previously identified barriers, this manuscript explicitly addresses several critical implementation challenges that commonly arise in interdisciplinary Public Health and Computer Science department collaborations. These include faculty workload pressures—often uncompensated in cross-departmental efforts—inter-departmental resource competition, institutional resistance to curriculum reform, accreditation constraints, and substantial variability in digital infrastructure and AI readiness, which are particularly pronounced in low- and middle-income settings (23). The framework also acknowledges sustainability risks once initial funding or enthusiasm diminishes, a common “*fizzle out”* pattern observed in many interdisciplinary initiatives. To mitigate these risks, the proposed approach embeds practical safeguards, including incentive realignment, sustained low-entry pilot projects, and reciprocal commitment mechanisms designed to maintain momentum using existing departmental resources.

By openly discussing these challenges, including the potential for optimism bias in bridge-building efforts, and by integrating targeted mitigation strategies, the framework enhances the credibility and realism of its recommendations. This balanced perspective underscores the value of the phased, reciprocal, and low-barrier design in promoting durable and context-appropriate Public Health and Computer Science departments' AI collaborations.

## 7 Example of successful public health-computer science departments integration across settings

While we described the status quo in most universities, there are bright examples that have pioneered the integration of computer science and public health with substantial success.

In high-income settings, several established initiatives demonstrate effective integration of public health and computer science. The Institute of Health Informatics at University College London was established in August 2014 with the mission to conduct high-quality, data-intensive research to improve health outcomes locally, nationally, and globally. It organically brings computer scientists and public health experts together to pursue projects that would not have been possible otherwise. Among other course offerings, the Institute has developed an “Artificial Intelligence Enabled” Master's in Research degree. Their interdisciplinary staff bridge fields including data science, bioinformatics, epidemiology, psychology, computer science and information governance, carrying out world-class research and providing students with the tools they need to carry out research in today's rapidly evolving field of health informatics (24). The UCL Institute of Health Informatics (United Kingdom) exemplifies this through joint research programs, dedicated data science MSc programs with a strong AI focus, and collaborative teams of clinicians and computer scientists (25). Additional prominent examples include the Johns Hopkins AI-X Foundry and Data Science Initiative, which foster interdisciplinary AI applications in public health and medicine, and the MIT Schwarzman College of Computing, which integrates AI across disciplines, including health (26). For broader global relevance, particularly in low-resource settings, networks from the Global South, such as the Global South Artificial Intelligence for Pandemic, Epidemic, Preparedness & Response (AI4PEP), a research network that brings together a network of researchers from Africa, Asia, the Caribbean, Latin America, the Middle-East and North Africa to explore health solutions that mobilize ethical AI to influence positive global health outcomes and IDRC-supported regional hubs, have implemented adaptive, equity-focused AI solutions for pandemic preparedness and public health challenges (27). Collectively, these examples illustrate how the proposed bridge-building strategies can yield tangible outputs and sustainable collaborations across diverse infrastructure levels, funding environments, and resource contexts.

## 8 Ensuring long-term sustainability

An important characteristic of the framework, as presented, is that whilst six steps (planks) exist, these must be jointly built on both sides of the divide by both Public Health and Computer Science academics. In doing so, a set of a dozen planks makes up a final full bridge, connecting the two sides for collaboration, but it's only if each side constructs its own planks that they will inevitably meet in the middle. This requires a combination of champions from each discipline, a passion for sharing knowledge, and an underlying infrastructure change that enables and rewards collaborative work. If this is achieved, then a preliminary bridge can be established, over which academics from both sides can walk to work with the others, bridging the divide.

But what of sustainability and maintenance? This is best accomplished by establishing a formal interdisciplinary center or institute dedicated to AI and Public Health, which can serve as a permanent hub providing ongoing administrative support, shared resources, visibility, and a clear identity for the partnership. Complementing this, a structured mentorship program should be created to pair junior faculty from both departments, fostering the next generation of collaborators through guidance, joint projects, and career development support. To keep the initiative thriving, the departments must regularly evaluate progress and adjust strategies based on faculty feedback as well as measurable outcomes such as the number of joint publications, secured grants, student involvement, and real-world impact.

### 8.1 Strategies for long-term sustainability

**Figure 3:**
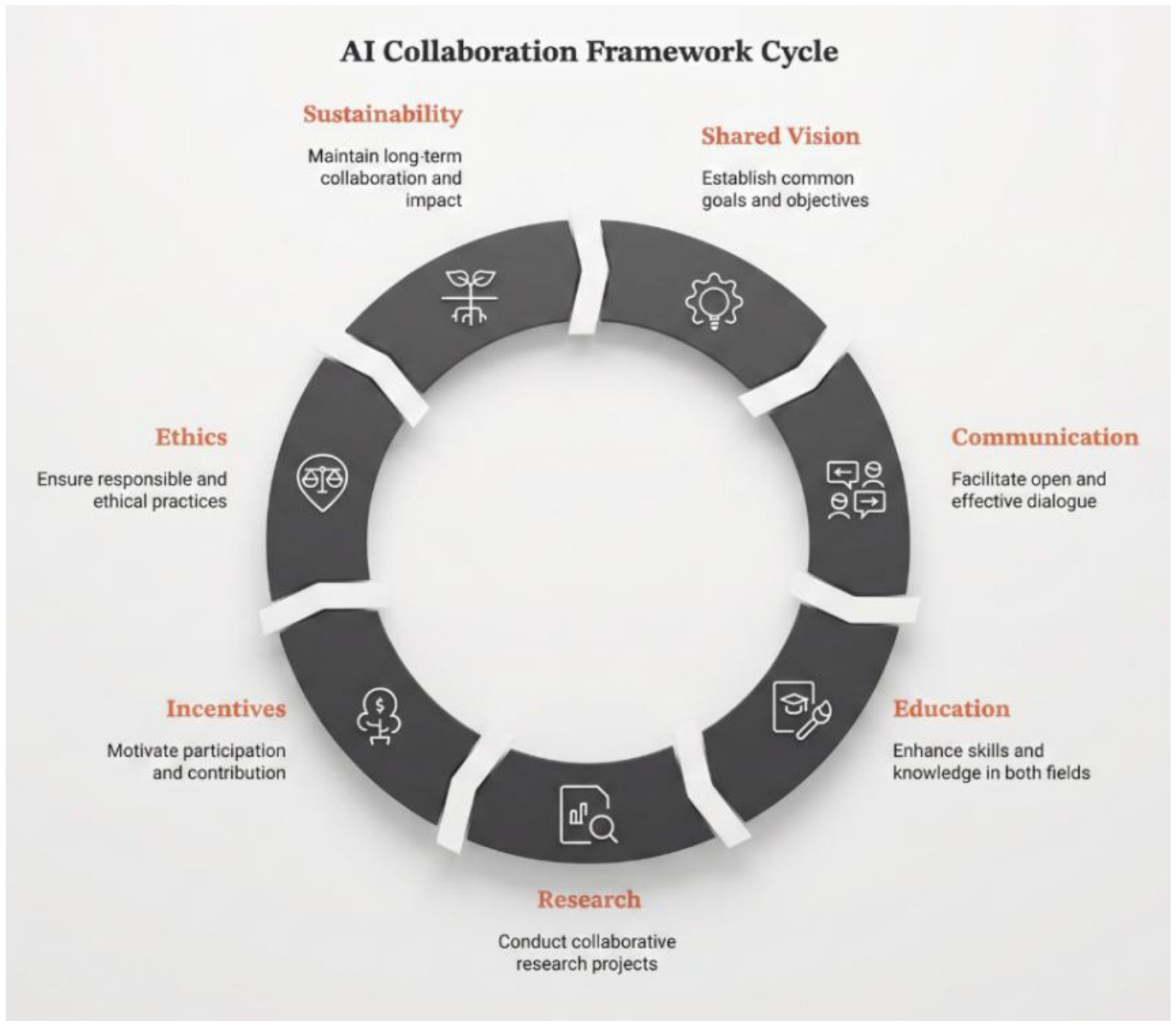
Continuing AI collaboration framework cycle.

For university leaders seeking to institutionalize Public Health and Computer Science departments' AI collaborations, robust governance structures, including the establishment of a formal interdisciplinary center supported by a joint steering committee with shared leadership between Public Health and Computer Science departments, pave the path for long-term sustainability. This is not a one-time endeavor but a cyclical one, where each step is bolstered by lessons learned (Figure 3).

Practical examples include organizing an annual symposium to showcase achievements, sharing lessons learned, and attracting new external partners, or establishing Communities of Practice (CoPs) that bring researchers together on a recurring basis to exchange ideas and sustain momentum (17). Complementary partnership strategies emphasize formal Memoranda of Understanding (MoUs), annual symposia, and recurring Communities of Practice to maintain momentum and knowledge exchange. Diversified funding models are detailed to ensure resilience beyond initial seed support. These include internal resource reallocations, targeted joint grants from government and the private sector, industry, and public health agency partnerships, and long-term endowments. Monitoring and evaluation mechanisms now feature regular KPI dashboards that track joint research outputs, student training metrics, and measurable real-world health impacts, supported by iterative feedback loops for continuous improvement, which helps to strengthen the path of sustainability. Institutional policy recommendations further reinforce sustainability through dual faculty appointments, revised tenure and promotion criteria that explicitly recognize and value interdisciplinary contributions, and structured mentorship programs for early-career researchers (28). Together, these enhancements transform the sustainability guidance into a comprehensive blueprint for converting initial enthusiasm and pilot activities into a self-reinforcing, enduring ecosystem. *By deliberately addressing the challenge of long-term sustainability*, this step transforms initial enthusiasm into an enduring, self-reinforcing, interdisciplinary ecosystem.

## 9 Conclusion

By addressing challenges through strategic collaboration, shared resources, and institutional support, Public Health and Computer Science departments can successfully build bridges to advance AI knowledge that is anchored in the cultivation of ethical, context-aware AI solutions that truly serve populations rather than merely advancing technology.

## Data Availability

The original contributions presented in the study are included in the article/supplementary material, further inquiries can be directed to the corresponding author.
